# Nonsense-mediated mRNA decay inhibition by HTLV-1 Tax protein

**DOI:** 10.1186/1742-4690-11-S1-O61

**Published:** 2014-01-07

**Authors:** Vincent Mocquet, Julia Neusiedler, Francesca Rende, David Cluet, Jean-Philippe Robin, Jean-Michel Terme, Madeleine Duc-Dodon, Christelle Morris, Hervé Le Hir, Vincenzo Ciminale, Pierre Jalinot

**Affiliations:** 1 Laboratoire de Biologie Moléculaire de la Cellule, Unité Mixte de Recherche 5239, Centre National de la Recherche Scientifique, Ecole Normale Supérieure de Lyon, Lyon, France; 2 Department of Oncology and Surgical Sciences, Institute of Neuroscience at the Department of Biomedical Sciences, Universita di Padova, Padua, Italy; 3 Institut de Biologie de l’Ecole Normale Supérieure, Unité Mixte de Recherche 8197, Centre National de la Recherche Scientifique, Ecole Normale Supérieure, Paris, France

## 

We have previously observed that degradation of mRNAs by the nonsense-mediated mRNA decay (NMD) pathway was affected in human T-lymphotropic virus type 1 (HTLV-1)-infected cells. This pathway is indeed strongly inhibited in C91PL, HUT102, and MT2 cells, and such an effect was also observed to a limited extent by the sole expression of the Tax protein in Jurkat and HeLa cells. In line with this activity, Tax binds INT6/EIF3E, which is a subunit of the translation initiation factor eukaryotic initiation factor 3 (eIF3) required for efficient NMD, as well as the NMD core factor upstream frameshift protein 1 (UPF1). Tax expression also alters the morphology of processing bodies (P-bodies), the cytoplasmic structures which concentrate RNA degradation factors. The presence of UPF1 in these subcellular compartments is increased by Tax, whereas that of INT6 is decreased. In line with these effects, the level of the phosphorylated form of UPF1 is increased in the presence of Tax. To further investigate how Tax can act on the stability of some viral and cellular transcripts which are prone to NMD, we examined whether Tax can directly bind such RNAs. By performing RNA immunoprecipitation analyses we indeed observed that Tax associates specifically with NMD-sensitive RNAs and the factors determining this binding are currently under investigation. These data further strengthen the notion that the effect of Tax on viral and cellular gene expression is not restricted to transcriptional control, but also involve posttranscriptional activities.

